# The Overfishing Problem: Natural and Social Categories in Early Twentieth-Century Fisheries Science

**DOI:** 10.1007/s10739-021-09655-4

**Published:** 2021-11-12

**Authors:** Gregory Ferguson-Cradler

**Affiliations:** Inland Norway University of Applied Sciences, Lillehammer, Norway

**Keywords:** Overfishing, Fisheries, History of science, Conservation, Commodification of nature

## Abstract

This article looks at how fisheries biologists of the early twentieth century conceptualized and measured overfishing and attempted to make it a scientific object. Considering both theorizing and physical practices, the essay shows that categories and understandings of both the fishing industry and fisheries science were deeply and, at times, inextricably interwoven. Fish were both scientific and economic objects. The various models fisheries science used to understand the world reflected amalgamations of biological, physical, economic, and political factors. As a result, scientists had great difficulty stabilizing the concept of overfishing and many influential scholars into the 1930s even doubted the coherence of the concept. In light of recent literature in history of fisheries and environmental social sciences that critiques the infiltration of political and economic imperatives into fisheries and environmental sciences more generally, this essay highlights both how early fisheries scientists understood their field of study as the entire combination of interactions between political, economic, biological and physical factors and the work that was necessary to separate them.

## Introduction

Perhaps no problem was as vexing for fisheries scientists at the turn of the twentieth century as the question of overfishing. When did overfishing occur? How could overfishing be identified? Were there different kinds of overfishing? What could be done to ameliorate it? The overfishing question was central to the new field of fisheries science and marked the fault lines around which research programs were initiated and institutions organized. While disagreements raged about the diagnosis, implications, and causes of overfishing, there seemed to be rough consensus along the lines of the definition given by Dutch fisheries scientist and senior figure in European fisheries research P. P. C. Hoek (1851–1914) in 1905: “Overfishing can be understood to be too severe fishing, and this means that more fish or better qualities of fish were taken away than natural production can replace” (Hoek 1905, p. xiii). In an 1894 article, Friedrich Heincke (1852–1929), a German biologist, similarly defined overfishing as the condition in which pressure on a population of fish was intense enough that the yearly production of new fish decreased, leading to a constant reduction in population (Heincke 1894). C. G. Johannes Petersen (1860–1928), head of the Danish Biological Station, in 1903 argued simply that overfishing was a decrease in the population of fish due to human activity (Petersen 1903). More frequently, however, early fisheries scientists discussed overfishing broadly, assuming its general outlines while papering over disagreements on its exact meaning and implications.

The assumption of a general definition was due in no small part to the fact that the term *overfishing* invariably implicated descriptive and normative conceptions of both natural and social worlds: it concerned the actions and interactions of fish and humans. The result was a scientific category that arose as a hotly contested assemblage of practices and styles of reasoning across social and epistemic boundaries. Natural science, the fishing industry, political economies of fish products, and geopolitics all influenced the ways in which the concept of overfishing was articulated and where it was identified to be taking place. It is no secret that, in the words of historian Carmel Finley, “fishing [is] always about more than fish” (2011, p. 81). Indeed, what would it even mean for fishing to be only about fish? Political, economic, and social interests are invariably deeply interwoven into the foundation of any sort of discussion or negotiation about fisheries. Fisheries science itself has seen the grafting and borrowing of models and assumptions from areas of knowledge as distinct as demography and insurance, economics, forestry, and physics (Smith 2007; Hubbard 2006, 2013, 2014a,b, 2018; Kingsland 1985).

In historical perspective, the development of fisheries science generally fits within a broader scholarship on quantification, statistics, and standardization in both history and sociology of science, and it is closely connected with the influential concepts of governmentality and *high modernism*. Theodore Porter (1995, 2012) has argued that new human sciences—particularly fields related to social, administrative, and economic management—came into being in the nineteenth century, portending a new basis for organizing social life. Society was to be grounded in rational thought instead of history and custom. In the context of democratic societies with burgeoning public spheres, a kind of valueless objectivity was sought to keep mass society at bay and provide efficient administration. An allied view, although different in chronology and mechanism, is that of Michel Foucault and related arguments of James Scott focusing on apparatuses of government to manage people and resources. Foucault highlighted an early modern philosophy of government whereby principles of wise management of the family and household—the economy in its original sense—were introduced into political practice to govern (Foucault 2000, p. 207). For Scott, eighteenth-century Prussian forestry was an example of the activity of a new sort of state that required mechanisms to survey its territory and population in ways that necessarily reduced complex, local, “thick” descriptions to synoptic, highly simplified, large-scale maps legible to an outsider. Representatives of the state thus reduced a blinding array of complexity to a small number of data points of state interest (Scott 1998).

In fisheries science, the drive to simplify nature by means of models devised through state-sponsored science with the goal of increasing and, indeed, maximizing catches has frequently had disastrous consequences for both humans and nature (Rozwadowski 2002; Cushing 1988; Taylor 1999; Bolster 2012; McKenzie 2010). Much of this literature is deeply critical of fisheries administration and postwar regimes of catch maximization and rationalization that sacrificed conservation for economic and geopolitical gains (Finley 2011; Hubbard 2014a,b; Mansfield 2004). Much work is grouped around the topic of *technocracy*, showing how fisheries science over the twentieth century incorporated progressively more quantitative and statistical techniques that gave the discipline an unhealthy overconfidence in the quality of its models, with tragic results as the models could not reflect the extreme non-linear complexity of fish population dynamics (Bavington 2010; Finlayson 1994). Furthermore, fisheries biologists and economists grew increasingly into their roles as *technocrats,* enmeshed in state apparatuses and cultivating a sense of superiority over the technically and mathematically unenlightened. Thus, they became more and more detached from everyday experiences of citizens (Cushman 2013). Many scholars have also found the connection of fisheries biology with economics unfortunate, showing how economists invaded the biologists’ turf, gained supremacy, and even rewrote the biologists’ history (Hubbard 2018). Accordingly, this is believed to have blinded fisheries scientists to the “economic ideals of efficiency, conservation, and modernization [that] have shaped fisheries biology and its goals” (Hubbard 2014a,b, p. 377).

More broadly, the idea of the *commodification of nature*, and, recently, neo-liberalization of nature, have become motivating topics of interest within the social sciences, activism, and larger public discourses that have been sharply critical of treating nature as just another economic object to be exploited within capitalist economies (Castree 2008; Telasca 2017; Merchant 1980; Worster 1977; Sandel 2013; Unmüßig 2014; Francis II 2014). The largest academic discussion on this topic has taken place in geography, based on Marxian and Polanyian approaches, including theories of accumulation, fetishization, and Polanyi’s notion of “fictitious commodities”: objects treated as commodities although they are not explicitly created for the market (St. Martin and Hall-Arber 2008; Mansfield 2004). The drive to “marketize everything,” again in the deeply influential Polanyian framing, triggers a “double movement” of social organization and resistance to expanding markets (Castree 2008, p. 144). This discussion comes frequently in response to “free-market environmentalism” that seeks to use market-based mechanisms for administration and protection of nature and natural resources, thus treating nature, according to this critique, as *only* of economic value (Anderson and Leal 2005; Andersen and Libecap 2014; see also Sabin 2013; Anker 2007).

Drawing on this literature, this essay focuses on the early twentieth-century history of *overfishing* as the key concept in the newly institutionalized discipline of fisheries biology. Fish, fisheries, and fisheries science have always already been political, economic, and social.^1^ I draw on debates in Western European fisheries science, particularly those that immediately preceded and subsequently took place under the auspices of the International Committee for the Exploration of the Sea (ICES), an international organization founded at the turn-of-the-century to further cooperation in marine research. Scientific, political, and economic understandings all congealed in fisheries science through specific practices, both physical as well as theoretical and analytical (Collins 1992; Warwick 2003; Shapin 2010). As this essay will make clear, separating biological and physical from social, economic, and political factors did not come naturally or easily.

## Geopolitics, Institutions, and Epistemology

In 1894, Friedrich Heincke, director of the German Biological Station on the island of Helgoland, published one of the earliest attempts to explicitly define and measure overfishing. Continuing the discipline-building and boundary work of the previous decades, Heincke began by staking the claim of fisheries science to the issue of overfishing. The question of whether overfishing was truly occurring in the North Sea, he wrote, “can only be resolved by comprehensive and scientific methods” (Heincke 1894, p. 1). Events of the last several decades had, he summarized, made research on overfishing vital and yet almost completely ignored. At the same time, the fishing industry on the North Sea had grown by leaps and bounds in its capacity to catch and to market fish (Heincke 1894, p. 5).

Technological innovations had fundamentally altered supply chains of fish products and led to booming household demand. Prior to the nineteenth century, fresh fish had been available for consumption on the coast, while further inland excessive transportation costs due to spoilage made it a “diet of the lord.” Traditional methods of preservation—included drying, salting, pickling, and curing—made transportation possible, though consumption was nonetheless limited in large parts of pre-industrial inland Europe (Teuterberg 2009, pp. 191–192). New technologies for preserving fish, as well as railroads and the means of quickly transferring the catch, made fish available to a much larger population and greatly increased demand. The canning technique of *full preservation* in the late nineteenth century allowed fish to be canned and retain their typical taste for up to a decade. Fresh fish could be preserved through artificial refrigeration and transported by rail quickly and cheaply to cities. The processing industry also began marketing partially prepared fish preserves that were already skinned, boned, and gutted (Teuterberg 2009, pp. 202–206; Tereshchenko 1917, pp. 13–14). Simultaneously, mechanization of the fishing industry had begun in the 1860s with the use of steam winches to haul up trawling nets. This allowed for nets of significantly greater dimensions to be deployed at lower depths and retrieved more quickly than those hauled up by hand. Nets that were lighter, did not entangle, and collected fish in a single cavity that could be emptied all at once rather than requiring removal of fish one-by-one, also increased capacity and cut down on required labor. By the end of the nineteenth century, many fisheries were dominated by steam-powered trawlers.

Skyrocketing demand and subsequent increased investment in fishing equipment created previously unimagined fishing capacity. The effect of this new technology on fisheries was far from certain for Heincke, though he noted that fishing communities in Helgoland were convinced that steam trawling was decimating local fisheries. This, he argued, was precisely the sort of claim that required the expertise and scientific knowledge of a newly professionalized science of fisheries. Heincke discounted the fishermen's knowledge of the seas—and their subsequent pleas for regulation of the fishing industry—in two ways. First, the distribution of the catch was unimportant to the general public weal, which was interested only in overall production. Even if it was true, as it clearly was, that small-scale fishers were harvesting smaller yields than previously, this in and of itself was of little concern. Local Helgoland residents had already largely shifted to other spheres of employment, he argued. Second, even if declining yields were occurring industry-wide, this was “by no means evidence of a reduction in overall fish stocks (*Bestände*)” (Heincke 1894, p. 7).^2^ Falling yields could have two causes: natural and artificial. By natural reductions, Heincke had in mind theories of herring migration, much discussed in his time, that sought to explain the well-known historical instances of abrupt and seemingly random disappearance and reappearance of herring in the waters of northern Europe (Smith 2007, chap. 1).

Artificial reductions were quite another thing. An increase in the numbers and capacity of predator populations could greatly reduce a population of fish. Given the fertility of fish—fisheries scientists often emphasized the ability of fish to produce untold thousands of eggs—a certain level of predation could be supported so long as a majority of fish were allowed to reach reproductive maturity. Under such conditions, a population of fish might decrease only slightly or not at all, while a similar amount of food (*Nahrungsmenge*) would simply be split up among a greater number of smaller individual fish. Danger would arise only when a sizable proportion of smaller fish was destroyed by predators such that a severely limited number reached the age of reproduction. “From here on out, the quantity of yearly-produced fry will steadily decline, which, despite the now unimpaired access to sustenance, will no longer suffice to keep the stock at its previous aggregate weight and with ever greater speed will lead toward complete destruction (*Untergang*). In our case we call this decrease in fish stocks 'overfishing*'”* (Heincke 1894, p. 9).

Despite his dismissive attitude to the general public (*Jedermann*), Heincke concluded that overfishing was, indeed, taking place due to inordinate destruction of fish fry by the German and English fishing industries, especially trawling that both harvested fish of all sizes and disfigured spawning grounds. From a political-economic (*volkswirtschaftlich*) standpoint, the expansion of trawling had increased the amount of nutrition available to the population, especially in Germany. But from the perspective of a “sea economy” (*meerwirtschaftlich*), trawl fishing represented an “irrational method, that in certain areas is becoming a fishery of plunder (*Raubfischerei*) in the full sense of the word” (Heincke 1894, pp. 11–12). He noted that what was needed were internationally coordinated regulations, which were already popular among coastal populations, especially in England, though frequently for the wrong, that is to say non-scientific, reasons (Heincke 1894, pp. 12–14). Throughout, Heincke emphasized overfishing as a *scientific* category, a framing he used to devalue knowledge of laypeople and appropriate expertise and influence for trained scientists.

Politically, the time seemed propitious for an international agreement. The word *overfishing* had first been coined in 1854 in British research commissions (Smith 2007, p. 117, chap. 3). By the turn of the century, it had been calqued into other languages, including German (*Überfischung),* Norwegian (*overfiske),* and Russian (*perelov*).^3^ The meaning of the word in different languages was roughly equivalent, which is to say, up for grabs. Most of the second half of the nineteenth century had featured government commissions, newly qualified fisheries scientists, and expeditions responding to complaints from local fishing populations frequently coming to the conclusion that there were no dangers of depletion and no need for regulation. By the 1890s, however, a number of states, according to Heincke, had accepted the need to research the question, and he considered eventual regulation to be a *fait accompli* following general agreement on the matter in negotiations at a 1891 international fishing conference in London (Heincke 1894, p. 2).

The next major international fisheries conference highlighted tensions between those who hewed to Heincke's notion of overfishing and others who saw in changing yields merely natural population fluctuations. The conference was convened in 1899 in Bergen, Norway, regarded by many as the “classical sea fishing country” (Decker et al. 1901, p. 3). Germany, Denmark (including Iceland and the Faroe Islands), Russia, England and Scotland, Finland, France (including Tunisia), Belgium, Austria, Romania, the USA, Sweden, and Norway (the latter two separately, despite being still formally unified) sent representatives to what was billed as the first of many future fisheries congresses. The occasion took place in the spirit of fin-de-siècle internationalism and optimism in the power of science to promote the common good.

At the same time, the proceedings showed a fault line in the young discipline, which the German delegates Heincke, Hermann Henking (1858–1942), and W. Decker, a Hamburg fisheries official, suggested was the unspoken third rail of the conference. “The increasingly burning question of overfishing of the sea and international protective regulations disappeared almost completely into the background because Norway, due to its particular situation and its mode of fishing … occupies a wholly isolated position and has for the moment little or no interest in this question” (Decker et al. 1901, p. 3). Indeed, the young but already dominant player in Norwegian fisheries science, Johan Hjort, spoke at the congress only of the importance of studying fluctuations and variations in yields and fish stocks in connection with environmental fluctuations such as the Brückner sunspot cycle (Brunchorst 1899, p. 126). In the end, the German delegates suggested that the congress might have benefited from avoiding the contentious issue. The time simply had not yet arrived for an objective, dispassionate (*objektiv, leidenschaftslos*) discussion. By their count, just one delegate had so much as motioned toward the issue when he briefly touched upon it during a presentation on the English steam-powered fishing industry (Decker 1901, pp. 4–10).

The Norwegian preference for explaining fisheries fluctuations as the result of natural cyclical variations—first in migration patterns and later in fluctuating population sizes—had begun in the 1860s with the investigations of Axel Boeck and Georg Ossian Sars on the two classic model species of Norwegian fisheries science: herring and cod. The assumption that herring migrated long distances, coupled with Sars's discovery that cod had free-floating eggs, led to the conviction that overall fish stocks must be constant. With such copious numbers of eggs produced every year in overwhelming excess of the number needed to regenerate the population, Sars argued that an identical number of fry would reach maturity every year (Schwach 2011, pp. 35–39). Instead, it was their location that was subject to change. Later, a different cause was identified: catches were directly related to potentially large, natural changes in fish reproduction from year to year (Schwach 2011, pp. 47–50). Explaining and predicting the causes of natural variations in fish populations remained the dominant question of Norwegian fisheries science and provided the model for management of fishing resources until the collapse of the Norwegian herring industry in 1968 initiated a reorientation of Norwegian fisheries science and management (Schwach 2011, p. 47; Smith 2007, pp. 11–14).

Many at the Bergen conference had called for greater international cooperation in fisheries research. As a result, a permanent body to coordinate international fisheries research, the International Council for the Exploration of the Seas (ICES), was founded in 1902. ICES was organized primarily as a body that would coordinate research and compile statistics. A short-lived central laboratory in Kristiania (Oslo), Norway, headed by well-known Norwegian scientist and explorer Fridtjof Nansen, worked to standardize measurements, methods, and scientific instruments, but ICES did not generally conduct research. While it could facilitate communication and common planning, it did not have any control over research vessels or facilities. Equipment and salaries of all but the small handful of core ICES officers were the financial responsibility of individual member states (Rozwadowski 2002, chap. 1; Smith 2007, chap. 4).

The organizational structure of ICES mapped onto the existing topography of theory and practice in ocean sciences. Hydrographers and biologists immediately went their separate ways and functioned in considerable isolation from one another for several decades (Rozwadowski 2002, p. 42). Within the biological section, two main committees were formed: Committee A, chaired by Hjort, to study migrations and natural fluctuations in fish stocks, and Committee B, tasked with overfishing. There was considerable overlap in membership of the two committees, although it is notable that there were no Norwegian delegates on Committee B. The two committees were pre-programmed to provide different answers to the same question: what was the status of fish stocks and why?

## Categorizing and Evidence: How to Study Fish

In his welcoming address to ICES delegates at the council's first meeting in 1902, Danish Foreign Minister Johan Deuntzer impressed upon the assembled delegates that it was time for marine science to move “from theory to practice*” *(Conseil Permenant International pour l'Exploration de la Mer 1902, p. 3). The fundamental goal of ICES was to address the practical concerns of people engaged in the economic exploitation of the seas. Since the late nineteenth century, governments both inside and outside the future ICES had increasingly looked to fisheries science for guidance in managing and exploiting fisheries. This created new impulses to theorize overfishing and to create, collect, and sort data that could be mobilized for scientific analysis. That neither theory nor data was prior to the other nor autonomous from one another will come as no surprise to historians, sociologists, and philosophers of science who have long been accustomed to notions of theory-laden data.

This section steps back from the immediate question of overfishing to look at the categories and evidence in the young science of fisheries. These were subsequently utilized in formulating notions of overfishing. Lorraine Daston has observed the difficulties of creating new categories in human populations where new statistical reference classes can only be formed if “one is first convinced that [their members] in fact possess enough commonalities to constitute a class, as opposed to a miscellany” (Daston 2008, p. 8). Categories cannot be created willy-nilly; they must be based on some logic. Subsequently, they have consequences for how we think about and order our world. As this section will show, social practices and concepts were deeply interwoven with those taken to be objectively “scientific.” This had clear implications for the kinds of evidence accepted and the categories and theories structuring fisheries science. As shown below, there could be no referent—fish or populations of fish—that did not involve humans too.

Categories in fisheries science formed through a process of communication between scientists, the fishing industry, and consumers. In *The Genesis and Development of a Scientific Fact,* Ludwik Fleck (1979) devoted considerable attention to communication between scientific experts, amateurs, and the general public. Fleck proposed his idea of the “thought collective” as an entity in existence whenever people exchanged thoughts, thus constituting a collective that “is transient and accidental, forming and dissolving at any moment” (Fleck 1979, p. 109). But, he also allowed that some collectives were far more stable, such as the medieval guilds. Stable thought collectives, he continued, generally consist of a small esoteric circle of experts and a larger exoteric circle surrounding it. Alternately, rather than concentric circles, constellations of thought collectives may also consist of any number of intersecting and overlapping circles. Inter-collective communication, he wrote, “always results in a change in the currency of thought” (Fleck 1979, p. 109). A change in the meaning of words communicated between collectives was unavoidable. Deborah Coen has insightfully argued that, for Fleck, the role of exoteric thought collectives—amateurs and the general public—was of vital importance in the production of scientific knowledge and “one of the most profound and little appreciated of his insights” (Coen 2012, p. 121). For Fleck, words and concepts were continuous but never stable in inter-collective communication and across the “gradation,” in Coen's turn of phrase, from esoteric to exoteric and the general public. Fisheries science provides the opportunity to think about communication not just between coherent thought collectives but between collectives of practice—scientists, fishermen, and participants in fishing economies.

In 1902, the first meeting of the scientific committees of ICES took place. In both Committee A and B, members moved to capitalize on the possibilities offered by ICES to construct a synoptic statistical picture of the northern European seas. Aware that the committees were yet untested and had no actual authority over the fisheries services and biological stations and vessels of the member-states, delegates of both committees made plans to use ICES as a central collection point for data. Data would be gathered in the same way as previously and forwarded to ICES to be collected by the chairperson (called the convener), with possible collation and standardization to come later. Each country would continue to use the gear and methods it had hitherto used. When Scottish representative D'Arcy Thompson suggested that an attempt be made to standardize the size and form of trawls used to collect samples, Henking and Heincke replied that this would not only be impossible but also undesirable given that “the experiments could not be ascribed any quantitative value” in the first place (Conseil Permenant International pour l'Exploration de la Mer 1902, p. 105). The value of such unstandardized statistics seemed, for the German delegates, to reside in the “qualitative” information given in the data—sizes of individual fish, weights, market values of the fish, geographic location of the catch, and so on. Inability to directly compare German and Scottish trawling data in a quantitative way was not at this point a problem for some ICES members.

The biologists in ICES then considered how statistics should be gathered. They first drew a line between biological data and commercial data. The Overfishing Committee outlined three ways in which fisheries researchers should obtain purely biological, “scientific” data that was not based on commercial yields. The first option was scientific trawling in fishing grounds. Such *trawling experiments* were designed to imitate commercial trawling. Equipment on scientific fisheries vessels was to be as similar to industry standards as possible. What made trawling experiments scientific was that scientists would conduct them with more precise records taken of geographic location and environmental conditions and more reliable counts of the fish hauled. Data points not usually recorded by the industry were also to be collected. A second method was to conduct experiments, as pioneered by C.G. Johannes Petersen, at the Danish Biological Station in Kattegat, the semi-enclosed body of water between Denmark and Sweden that forms the straits between the Baltic and North Seas. By this method, attendants at biological stations caught fish, threaded a metal wire attached to an identification tag through the fish's undersides, and released them. Promise of a monetary reward was clearly indicated on the tag to encourage fishermen to return them promptly in case of capture. Fishing intensity, too, could be estimated by calculating how many released fish were caught and how quickly. Third, egg-counting methods initiated in the 1880s by Victor Hensen in Kiel could be used to make inferences about population numbers (Mills 1989, chap. 1; Lussenhop 1974). Beyond these scientific methods, ICES scientists also used statistics from the fishing industry. These had the major weakness of being recorded by fishermen who lacked the rigor and precision of scientists and trained assistants and did not always use the data points that biologists sought, such as time and place of catch. On the other hand, they had the advantage of being numerous. While ICES scientists could be dismissive of data gathered by fishermen, most also recognized that industry data was essential and that the cooperation of fishermen would be needed and could generally be counted upon (Conseil Permenant International pour l'Exploration de la Mer 1902, p. 187).

Biologists made some attempts to record industry statistics according to their own preferred scientific categories. A Scottish representative in 1903 noted that, in Scotland, statistical officers worked aboard many fishing boats to record information such as number of hours actively engaged in fishing and exact catch locations. Based on this, scientists could then create charts divided into squares of one-degree of latitude and longitude showing the abundance of fish derived from catches and total fishing time. Such detailed mapping, however, involved a statistical officer making separate inscriptions on top of industry data and was more expensive than most fisheries research institutions could afford. Henking reported that he too had attempted this but had given up making such geographically accurate charts. Instead, he found it more expedient to return to the usual practices of making charts divided into the geographical categories known and used by fishermen (Conseil Permenant International pour l'Exploration de la Mer 1902, pp. 107–108).

Much more frequently, industry data were translated or simply imported wholesale into the biologists’ statistics. Fundamental categories were sometimes adopted from the fish trade with little debate. Length, for instance, was recorded by industry according to market categories—small, medium, and large. These categories were widely used in fisheries science as well. When these categories changed in Britain in the first decade of the twentieth century, and the category of *extra small* was included in market prices, commercial statistics duly reflected the change. Writing an article on statistical analysis several years later, Bjørn Helland-Hansen, a mathematically-inclined oceanographer assisting with biological statistics, prefaced his study by clarifying that he was only dealing with biological statistics, that is, data gathered on fisheries research vessels, not trade data. But while his length data were all in centimeters, he calculated the market sizes, including the new *extra small* size, and used the market categories throughout his presentation and analysis of the statistics, thus importing and translating scientific data into commercial categories (Helland-Hansen 1909). Clearly, many seeking a scientific approach to fisheries still thought in the categories of the fisheries industry.

Scientists were aware of the different categories of data and were often intent on keeping them separate. There was, nonetheless, a considerable gray area between the two, and it was not always easy to say if a certain category was a scientific or industry standard. At the first meeting of ICES in 1903, delegates worked through the implications of size-divisions that scientists had inherited from industry. Heincke noted that in Germany the commercial sizing distinctions essentially coincided with the biological distinction between sexually mature and immature fish. Dutch delegate and ICES General Secretary Hoek disagreed; it appeared to him that the committee was conflating two very separate categories and things, “the immature fish of the biologist and the undersized fish of the fisherman” (Conseil Permenant International pour l'Exploration de la Mer 1903, p. 135). The committee chair and coordinator of the statistical collation effort, Walter Garstang of Great Britain, quickly replied that he “was of the opinion that the study of the two problems could practically not be separated” (Conseil Permenant International pour l'Exploration de la Mer 1903, p. 135). For Garstang, one of the most senior members of the committee, this most important of markers in the life cycle of fish—the threshold of maturity and marketability—simply could not be disentangled into scientific and industry categories. It was indelibly both.

Thus, commercial and biological data and categories intermingled virtually inseparably in the early decades of fisheries science. Even those who did not want to use industry data found it impossible to ignore completely. Others thought the task of disentangling them was impossible, while still others believed using commercial and political categories would be beneficial to producing worthwhile knowledge.

## What is Overfishing?

**Fig. 1:**
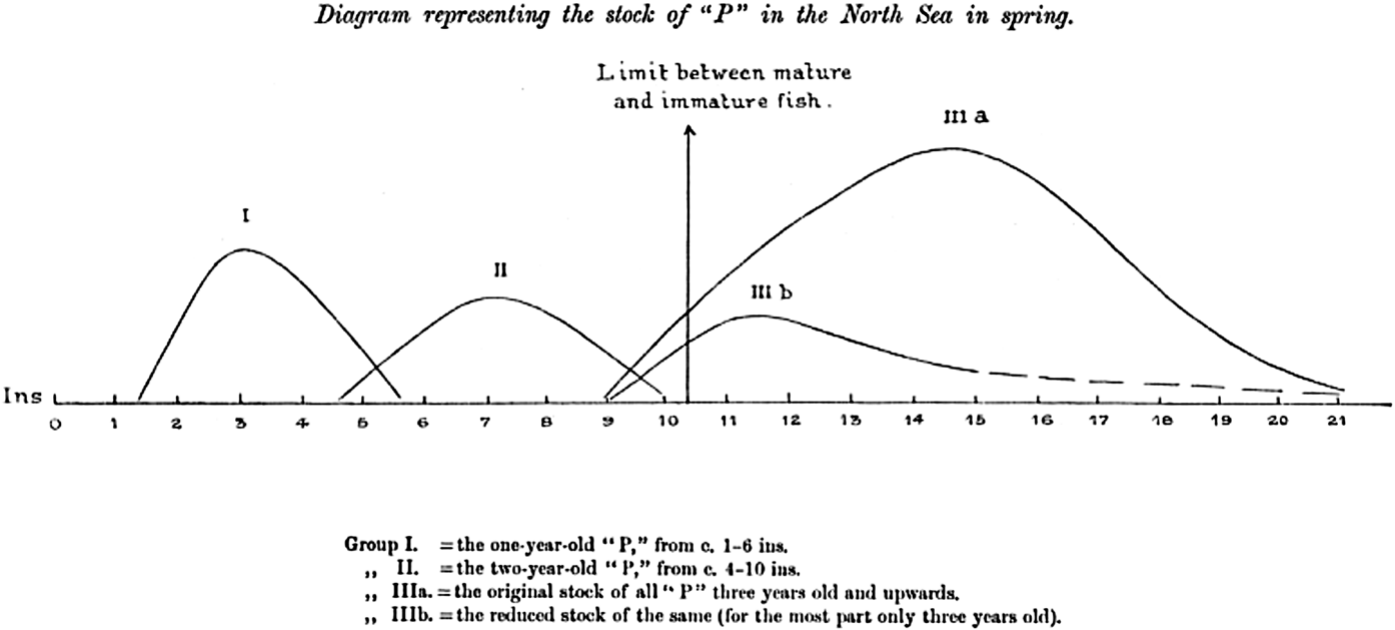
Petersen’s graphic model of fish populations based on length (on the horizontal axis), population size (on the vertical axis), with size dividing mature and immature fish clearly marked (Petersen 1903, p. 595)

In ICES's first year of existence, Committee B member and Danish Biological Station head Petersen published an article descriptively and provocatively called “What is Overfishing?” in a British fisheries journal. The paper set up the following hypothetical situation. Imagine, Petersen wrote, a biologically self-contained area of sea in which statistics suggest the value of a total yearly catch is decreasing with every year. Fishing intensity and price of commodities remain unchanged. The physical conditions have remained constant, or at least not changed unfavorably. If the decrease in yields can be ascribed to humans, he wrote, overfishing is taking place. Take, then, another “hypothetical,” that will be called fish “P” (whose characteristics just happened to be identical to the Kattegat plaice). As has been observed with plaice, the total yields and average length of “P” have been decreasing yearly. Is overfishing occurring? It depends. Perhaps this was due to newly initiated fishing of accumulated stocks that had not been hitherto subjected to industry. “Annual decreasing catch is not strictly an example of over-fishing, at any rate it is only an exceptional kind of overfishing, which is inevitable, and to some degree desirable,” he argued (Petersen 1903, p. 588). But there were three other kinds of possible, less-desirable overfishing: overexploitation of mature fish that impaired reproduction at stable levels, fishing that reduced the overall size of fish such that they were no longer “sufficiently saleable,” and the destruction of immature fish, which had no market value in any case, as the by-catch of another industry (Petersen 1903, p. 591). Market conditions and prices were central and essential to the analysis; without them, the entire concept lacked coherence. Further, overfishing was not inherently bad or good; it was both unavoidable and sometimes desirable that such fishing happen (Fig. 1).

Another approach to the overfishing problem came in an article authored a year later by a young British biologist Harry Kyle. Like Petersen and Heincke, Kyle sought to disentangle the question of overfishing from that of decreasing yields. Just as decreasing yields do not indicate overfishing, overfishing could be present, he argued, even during periods of increasing yields. Kyle's major argument, however, was to focus on overfishing as a “practical and economic problem, which has to be measured not in terms of quantity but in terms of value, i.e., of the capital employed and income earned” (Kyle 1905, p. 4). His study was squarely based on statistics in hand rather than Petersen's studiously maintained hypothetical. The statistics that he was most interested in were those with monetary value of the fish built-in—not the total catch per boat per day but rather total value of catch per day. The question depended on “the most economical boat, gear, mode of working as well as the demand for fish in the country” (Kyle 1905, p. 15). Kyle's version of overfishing was not directly linked to populations of stocks or yields but was a relationship of the quality (understood as size expressed in either weight or length of individual fish) and quantity of aggregate yield in market prices. Theoretically, he suggested, one could mathematically measure precisely when overfishing began, but this calculation would have to be constantly updated to track changing market prices for fish. In short, overfishing could be said to be present “when the average catch had so diminished all around that it no longer paid the fishermen to work” (Kyle 1905, p. 15).

Kyle then sought to mediate between varying arguments as to whether the Kattegat plaice fishery was in decline. To do this, he separated “the overfishing aspect” from the “biological or scientific aspects of the matter,” which is to say the population dynamics of fish stocks (Kyle 1905, p. 20). Evaluating a claim of Petersen's that the Kattegat plaice fishery had reached its peak in the 1890s and had been declining ever since, he cross-tabulated total plaice yields of the previous decade with average prices per kilogram to calculate total yields of plaice by weight. The complications of making such calculations were numerous: the exact origin of the catch was not always clear, plaice statistics might include a small and variable portion of other fish, and so on. For the Danish side (Kattegat is a shared fishing ground between Denmark and Sweden), the total yield was obtained in Danish kroner, which then had to be converted into weight. Average historical conversion factors, however, were available to convert only between value in Danish kroner and quantity, not average or total weight. Another set of historical conversion factors was needed to convert length to weight because the average weight had been decreasing—from 7.5 kg per score of fish in 1889 to 6.25 kg in 1903. Each conversion contained considerable uncertainty. His conclusion was that the size of fish had decreased, prices had increased, and fishing had intensified, but total yearly yields in weight remained constant. There was a difference, then, between depletion of fish stocks (a biological measure) and overfishing (a biological/socio-economic category). One did not necessarily imply the other. Here, depletion had not happened—aggregate yields remained steady. Was this a case of overfishing? “In the case of the Kattegat plaice fishery, it may be said, that the amount of fishing has been practically overfishing, or at least within the borders of overfishing, for a number of years. In other words, overfishing may result in the decrease of fish and a cessation of a fishery, or it may not” (Kyle 1905, p. 24). Overfishing was, at best, a notion of considerable plasticity.

Added to the amorphousness of the concept of overfishing, Kyle suggested that even a fishery that was not being overfished might be irrationally managed. Large plaice were worth some three times more per unit of weight than were small plaice. In this case, why not harvest the same aggregate weight in only large fish? Furthermore, he argued that it was precisely the increase in fishing that led to a decrease in size and, thus, a further decrease in cost of small fish. Why increase fishing effort only to drive down the price? This was “doubly irrational” (Kyle 1905, p. 58). Far from their seemingly stable definitions, in practice overfishing and rational fishing were moving targets. Fisheries scientists agreed on some, but not all, factors that needed to be considered to account for overfishing. Different people privileged different indicators, statistics, and theoretical constructions.

Since the first meeting of Committee B on overfishing, several delegates to ICES had noted the concern of their governments that focusing on overfishing was unlikely to lead to practical solutions to declining yields. Hoek noted that the Dutch government was uneasy about the committee for this very reason, while Henking denounced the “impracticality (*Unzweckmässigkeit*) of the unfortunately overused word 'overfishing'” (Conseil Permenant International pour l'Exploration de la Mer 1903, p. 135). Both suggested that, in the context of ICES, the overfishing question was not the right one to ask. In the following years, other voices joined in the chorus that overfishing was simply too complex. In addition to this was the practical political problem of funding. ICES had formed for a provisional three years, after which member governments expected to see practical results. Delegates on the Committee decided that it would be wise to shift the stated goal of the Committee to a topic in which obvious progress could be made in a short period of time. The Overfishing Committee became the Plaice Committee.

There was also a split in the ICES bureaucracy between those privileging big commercial data sets and the smaller, but presumably more accurate and reliable, data created by trawling experiments, tagging, and egg counting. Again, this was a preference that had intertwined economic, political, and scientific aspects and implications. Those like Petersen—the majority in Committee B—preferred “scientific” data. Indeed, while Committee B had initially been responsible for collecting trade statistics, this duty had been spun off to the ICES Bureau, the permanent staff of the Council, consisting of the President, Vice-President, General-Secretary, and scientific staff: the physicist-hydrographer Martin Knudsen and biologist Kyle. Kyle was also a member of Committee B as a delegate from the UK. In 1907, delegates of this committee announced that they had collected a sizable portion of “scientific” data resulting from a relatively new method of determining the age of fish—counting the calcified rings of the otolith, a bone in the inner ear of fish, just as one would count the rings of a tree. Heincke announced that he had compiled data from over 6000 otolith readings, a particularly large sample. Kyle argued that such data should be curated by the Bureau rather than Committee B, given that the Bureau specialized in methods of investigation dealing mathematically with large sets of data. This finding was swiftly dismissed by committee chair Garstang, who significantly out-ranked Kyle, and a compromise position was reached that the data would be shared by the Bureau and the committee (Conseil Permenant International pour l'Exploration de la Mer 1906, p. 22). But behind this quick compromise stood a fuzzy and negotiable boundary, bureaucratic and epistemic, on who would administer what sort of data.

Looking back in 1928 on the first years of ICES, Kyle argued that only large data sets gathered from across ICES and recorded in standardized format could have led to definitive answers to the overfishing problem. He recounted how, when ICES was founded, biologists preferred to work in small areas on strictly delineated problems, and “the wide synoptic view which international statistics give was foreign and unreliable” (Kyle 1928, p. 1). After a year-long effort that culminated in a statistical compilation by Hoek and himself in 1905, Kyle claimed that biologists were at last convinced that statistics could and should be the single-most important part of international cooperation. However, since that time, international efforts to collect comparable cross-country statistics, including average weight and place of catch, had failed. Moreover, statistical summaries reported by individual countries had declined in quality, demonstrating that biologists once again doubted the power of international statistics to solve major problems in fisheries science. Kyle recounted in detail the failure of the ICES central bureau to coordinate an international effort to measure fish in fish markets in an attempt to discern if average size of caught fish was decreasing, a key data point in the overfishing question. In Kyle’s telling, the blame lay squarely on England for refusing to participate (Kyle 1928, pp. 1–4). Disregarding the subjective nature of this account, it testifies clearly to the continuing tension in fisheries science over what constituted useful and practical evidence.

By the late 1920s, the problem of overfishing seemed to have been reconsidered and rearticulated as not so much the guiding question of fisheries science, but as simply the outcome of irrational management of fisheries. Earlier scientists had called attention to decreases in numbers of large fish in yields, in total yields, in sizes of average catch per boat per outing, or pointed to other supposed indicators of overfishing. By 1928, Kyle argued, a second phase had begun. The last decades had shown that levels of fishing had remained roughly similar, even after greatly reduced exploitation during the war. This did not seem like overfishing. “Clearly, the word also contains a hidden bias, which hinders the calm, non-partisan consideration of past events. In fact, we should more properly speak of fisheries of greater or lesser intensity and study the consequences comparatively” (Kyle 1928, p. 29). Overfishing, for Kyle, was not the right category of analysis at all; instead, he seemed to be suggesting something akin to the collective strategy of the 1899 Bergen conference, where overfishing as an explicit topic was taken off the table, allowing agreement and discussion over other issues to proceed.

Finally, Kyle tackled a perennial problem in the plaice fishery: the problem of the small plaice, notably the same pivot made by ICES several decades prior. In an analysis bolstered by, but not dependent on, statistics, Kyle considered numerous data points and multiple possible explanations before reaching a detailed conclusion to the problem, based on both quantitative and qualitative evidence, and suggesting measures for amelioration. Twenty-five years earlier he had suggested that, theoretically, overfishing, at least in a biological sense, could be calculated using a handful of the most important fishing statistics. By 1928, while ruing the lack of statistical data, he was considerably more skeptical that natural processes could be reduced to a few numbers.

A different conclusion, albeit using an allied approach, was forwarded by E. S. Russell, a Scottish fisheries scientist active in ICES who would later become a significant philosopher of biology known for holism and support for Lamarckian genetics (Graham 1954; Roll-Hansen 1984). In an influential 1931 article entitled “The Overfishing Problem,” Russell, like Kyle, highlighted the extremely complex and variable conditions that must be considered in a discussion of overfishing. His, too, was to be a non-mathematical description of the problem. “It is my aim here,” he wrote, “to formulate in a simplified and general way, and without mathematical treatment, the broad facts of the case, to state in simple language those elementary principles that are at the back of everyone's mind who deals with the problem of the rational exploitation of fisheries” (Russell 1931, p. 3).

Russell approached a fishery through the lens of accounting. He began with a simple hypothetical model of an ideal fishery. Consider, he posited, a self-contained stock of fish of a particular species and population forming an exploited fishery. Fish bigger than length *l* would be caught by fishing nets. What, he asked, would occur to the total weight of catchable stock *S* over one year of fishing? He duly divided the changes into “credit” and “debt” sides of a balance sheet. The total weight of all young fish surpassing length *l* (denoted by the variable *A*) would be one source of credit. The catchable stock not caught during the year would continue to grow, adding *G* amount of total weight to the stock. On the debt side, the total weight of all fish caught (*C*) would need to be subtracted, as would the weight of all fish dying from other causes (*M*). The total weight of the fishery *S*_*2*_ would be: *S*_*2*_ = *S*_*1*_ + (*A* + *G*) − (*C* + *M*). “We start with working capital S_1_; to this is added in the course of a year (*A* + *G*), and from it taken away (*C* + *M*). At the end of the year our working capital is *S*_*2*_, which will be greater than, equal to, or less than *S*_*1*_, according as income (*A* + *G*) has exceeded, equaled or fallen below expenditure (*C* + *M*)” (Russell 1931, pp. 9–10). Though expressed in terms of an accountant, such a presentation was not intended as a means for actually modeling a fishery but as a tool to think about natural resource use. The equation suggested the type of “thin,” synoptic description of high modernism discussed by Theodore Porter (2012), but every variable was “thickly” described—at length and in individual detail. Russell used this balance sheet to elucidate his understanding of rational exploitation. The aim of a rational fishery, he argued, was to maximize the annual yield (income) in a way that did not decrease carryover of capital from year to year. This was a financial view of nature, but with aggressively conservationist implications.

In her 1966 book *Models and Analogies in Science,* Mary Hesse argued that models and metaphors are fundamental to the process of scientific cognition. They connect systems or objects that have some shared traits (de Chadarevian and Hopwood 2004). Metaphors and models are made up of positive, negative, and neutral analogies: positive analogies are properties of the model known to correspond to observed entities (the “real” world), negative analogies are those that are known not to correspond, and neutral analogies are properties of the model about which not enough is known to posit their existence or absence in the world. For Hesse, the real power of models is in the neutral analogies, which can be productive by pushing scientists to explore their object of analysis in ways that might or might not hold (Hesse 1966). In fisheries science, however, the financial and economic metaphors were deeper than this. Interest on capital might have been a neutral analogy to the production of new biomass from a stock of fish. But this was more than simply an analogy because, for many, fish *were* in fact capital. This is perhaps why banking and balance-sheet accounting proved to be such resilient metaphors in fisheries science. It is also worth noting that precisely these metaphors were models for the most conservative and conservationist approaches to fisheries management. As observers of environmental science and administration, science studies scholars and environmental social scientists and humanists must be aware of both the creative flexibility and new perspectives models afford, as well as the dangers of complete or one-sided unbalanced monetization and utilitarianism.

## Conclusion

In focusing on the practices of early fisheries scientists as they struggled toward a coherent concept of overfishing, this essay has emphasized the indelible interconnections between science, industry, and the market for fish products. How data was collected, what data was collected, how overfishing was theorized, and how fisheries were modeled were all assemblages of practice communicated across socio-epistemic boundaries. Statistically inclined biologists disagreed with field biologists about what sort of data constituted useful evidence. The concept of overfishing, too, was malleable beneath the most general notion that overfishing represented some sort of decrease in fisheries. What sort of decrease—in stocks, yields, monetary values, or profits—was still up in the air, although by the 1920s and 1930s scientists from across Europe, in both capitalist and socialist economies, increasingly attempted to crack the overfishing nut in terms of profit-maximizing rationality.

Much of the literature in environmental social sciences and humanities bemoans the infiltration of the political and economic imperatives into fisheries and environmental science and conceptions of nature more broadly. Whether it is the abstract mathematical models of population biology and fisheries economics, the prestige of academic economists, or geopolitical considerations, numerous scholars have depicted postwar fisheries and environmental sciences as being won over by technocracy and strictly economic conceptualizations of rational resource management. Economics and politics came to be prioritized over biology. Focusing on the first part of the twentieth century, this essay suggests that the distinction between these two domains—at the level of concept, modeling, and data collection—was, at best, murky for early fisheries biologists. Fish were—and are—both natural, biological beings and economically valuable potential assets. To that extent, attempts to push economics and politics out of fisheries biology for the sake of purely biological conservation models is to argue for a purity of fisheries along the lines of the concept of “wilderness” influentially critiqued by Cronan (1996) and White (1996). Just as this is not to deny that the natural world has value outside of its human utility, so too is it to recognize that as long as *Homo sapiens* have walked the Earth, they have been a component part of it. It seems reasonable that the models of fisheries science would, and should, reflect the biological, physical, economic, and political.

This account invites science studies scholars who examine fisheries to drill down and examine at a deeper level how these are infused in the practices, both physical and mental, of the science, and how scientific categories and objects are constituted and reconstituted. This entails a deeper and more explicit understanding of the models in use and how they are constructed and mobilized. There can be no final right answer in what the right measure of science, politics, economics, and social considerations should be in fisheries models. They reflect changing natural and social realities and norms. In the end, perhaps this is the crux of the matter. The categories and concepts of our present world are deeply ingrained in fisheries science. To reimagine economies and fisheries in a more equitable, conservationist, community-based, or democratic manner—an abiding and no doubt worthwhile goal—will take a science of fisheries that fully combines the study of the interactions between the physical, biological, and human worlds.

## Footnotes

1 In some sense, fishers, fisheries scientists, and administrators have long been aware of this. The word *fishery* as used by actors in this paper through the present usually denotes both a group of fish and the humans who harvest them delimited by some mix of geography, biological characteristics, and means of human exploitation or human use. It inherently involves both fish and humans, rather than simply a population—or stock—of fish, on the one hand, or just fishers, the fishing industry, and fish producers, on the other. I retain this usage here. McEvoy (1990) and Bavington (2010) are two of the best works of scholarship treating fishing from all these angles.

2 The concept of a *stock* is also one with a history. Similar and at times interchangeable with the concept of population, according to Sinclair and Solemdal (1988), “stock” also included emergent concepts of different races of fish, although this aspect goes unanalyzed there. Twenty-first century fisheries biologists credit Heincke for the first use of “stock” to refer to subgroups of a species possessing different physical traits (Booke 1999). Here, too, linguistic differences pointed in different directions. The German *Bestand* suggesting both inventory—along the lines of the English—as well as, in its financial meaning, *portfolio*, rather than individual share as in the English. To select just one more example, the Russian *zapas* (reserve) also has financial meaning, among others. For more on the history of the concept, see Bavington (2010) and for general remarks Telesca (2017).

3 In the diplomatic French used at international conferences and in multi-national documents, it remained, however, *la diminuition du rendement de la pêche.*
